# A Hybrid Oleic-Acid-Derived Polymer Electrolyte Integrating Single- and Dual-Ion Conducting Systems for Lithium-Ion Batteries

**DOI:** 10.3390/polym18060773

**Published:** 2026-03-23

**Authors:** Wansu Bae, Sutradhar Sabuj Chandra, Doyul Lee, Donghoon Kang, Hyewon Na, Jiye Lee, Hohyoun Jang

**Affiliations:** Department of Energy Material Science, Konkuk University, 268 Chungwon-daero, Chungju-si 27478, Republic of Korea; gelp621@naver.com (W.B.); chandra@kku.ac.kr (S.S.C.); b6769@naver.com (D.L.); rkdehdgns28@naver.com (D.K.); gpdnjs3z3@naver.com (H.N.); wldp9902@naver.com (J.L.)

**Keywords:** polymer electrolytes, in situ polymerization, hybrid ion conduction, bio-based materials, lithium-ion batteries

## Abstract

In this work, a hybrid polymer electrolyte integrating single- and dual-ion conducting systems was developed for lithium-ion batteries using bio-based materials, namely oleic-acid derivatives and epoxidized soybean oil, through an in situ polymerization process. The fixed FSI anions in LiEFSOA enhance the selectivity of Li^+^ transport, while the cross-linked network formed by ESO provides mechanical stability, and the LiFSI incorporated into the polymer matrix helps maintain sufficient overall ionic conductivity. In addition, the long C18 oleic chains increase the internal free volume of the matrix, thereby improving segmental mobility within the amorphous phase. The in situ polymerization inside the cell causes intimate interfacial contact between the electrode and electrolyte, achieving an ionic conductivity of 1.05 × 10^−4^ S cm^−1^ at 30 °C. Electrochemical evaluation using LiFePO_4_/FSOA-2/Li cells shows an initial discharge capacity of 149.09 mAh g^−1^ and a capacity retention of 81.09% after 100 cycles, and the average coulombic efficiency was 99.62%, demonstrating that the designed FSOA electrolyte exhibits stable cycling performance and competitive capacity. Overall, the combination of eco-friendly materials and a hybrid ion transport strategy provides a promising platform for developing sustainable and high-performance polymer electrolytes for lithium-ion batteries.

## 1. Introduction

A lithium-ion battery (LIB) is a vital energy storage technology with a wide range of applications, including electronic devices, electric vehicles, and energy storage systems (ESSs) [1,2]. Their development requires high ionic conductivity, electrochemical stability, and high working voltage [3,4,5]. However, current commercialized liquid electrolyte-based LIBs have recently had problems such as flammability, electrolyte leakage, and poor thermal stability [6,7,8]. Polymer electrolytes, which have high thermal stability and mechanical strength, are attracting attention as an alternative to address these issues [9,10,11,12]. Despite these advantages, the most widely studied polyethylene oxide (PEO)-based electrolytes exhibit inherent limitations, including low ionic conductivity caused by electrode–electrolyte interfacial resistance, high crystallinity at room temperature, and concentration polarization arising from a low lithium transference number (t^+^ ≈ 0.4) [13,14,15,16,17,18].

To overcome these performance limitations while reducing dependence on fossil-derived resources, increasing attention has been directed toward the design of sustainable electrolytes. Bio-derived raw materials such as vegetable oils and cellulose provide promising advantages owing to their abundance, biodegradability, renewability, and facile chemical functionalization [19,20,21,22,23]. Oleic acid and epoxidized soybean oil (ESO) are particularly attractive as polymer electrolyte precursors, as the presence of carboxyl and epoxy functionalities enables a wide range of structural modifications [24,25,26,27].

Liquid electrolyte systems move lithium ions through anions and electrolytes [28,29,30], while ion transport mechanisms in polymer electrolytes can be broadly divided into two types of pathway. The first is segmental relaxation, which enables lithium ions to coordinate and decoordinate repeatedly with polar functional groups in a polymer [31,32,33,34]. The second is a hopping mechanism by which lithium ions jump and move through anions immobilized on the polymer. Conventional PEO-based electrolytes with lithium salt primarily rely on segmental motion, resulting in relatively high ionic conductivity but low t^+^ values. Single-ion conducting polymer electrolytes (SICPEs) introduce immobilized anions to enhance lithium-ion selectivity, resulting in high t^+^ values but relatively low ionic conductivity [35,36,37]. Therefore, to develop electrolytes with high ionic conductivity and lithium transfer numbers, a hybrid strategy is needed that simultaneously achieves polymer chain flexibility and selective lithium transport via immobilized anions.

One practical approach to implementing such hybrid strategies is the use of epoxy groups, which enable in situ cross-linking polymerization during electrolyte fabrication. This polymerization minimizes the interfacial gap between the electrolyte and the electrode in the precursor stage, while allowing polymerization to occur directly within the system, thereby creating smooth contact surfaces that facilitate lithium-ion transport [38,39,40,41]. Monomers with a cyclic ether structure, such as epoxy or 1,3-dioxolane (DOL), offer the advantage of enabling cationic ring-opening polymerization (CROP) without an additional initiator, as the lithium salt acts as a Lewis acid [42,43,44], and the resulting ether linkages (C–O–C) promote segmental relaxation [45]. Compared with externally fabricated polymer films, this approach reduces interfacial resistance and enhances both ionic conductivity and cycling stability [46]. Moreover, epoxy-based in situ polymerization using bio-derived precursors such as fatty acids or vegetable oil derivatives provides a pathway to simultaneously achieve high electrochemical performance and sustainable material design.

In this study, we developed a polymerized hybrid electrolyte (FSOA) using an in situ method, incorporating lithium epoxidized fluorosulfonyl imide oleic acid (LiEFSOA) synthesized from an oleic acid derivative as a functional monomer and utilizing ESO as a flexible matrix. This system combines the single ion conducting function of LiEFSOA with the structural flexibility of ESO, resulting in enhanced ionic conductivity, a high lithium transfer number (t^+^ ≈ 0.62), and high oxidation stability (≈ 4.8 V). In this study, we suggest the possibility of designing a hybrid polymer electrolyte based on renewable resources and present a new strategic approach for next-generation eco-friendly lithium-ion batteries.

## 2. Materials and Methods

### 2.1. Materials

Oleic acid (OA, >99%) and lithium hydroxide monohydrate (LiOH∙H_2_O) were sourced from Sigma-Aldrich (Seoul, Republic of Korea). Chlorosulfonyl isocyanate (CSI, >98%), antimony(III) trifluoride (>99.8%), hydrogen peroxide solution (30 wt% in H_2_O), and acetic acid (glacial, ≥99%) were also obtained from Sigma-Aldrich (Seoul, Republic of Korea). Amberlite^TM^ IRC-120(H) was ordered from Thermo Fisher Scientific (Waltham, MA, USA) and lithium bis(fluorosulfonyl)imide (LiFSI, high purity, >99.9%) was provided by EPchemtech (Gunsan, Republic of Korea). Solvents such as ethylene carbonate (EC, ≥99%, H_2_O < 10 ppm, acid < 10 ppm), diethyl carbonate (DEC, ≥99%, H_2_O < 10 ppm, acid < 10 ppm), and N–methyl–2–pyrrolidone (NMP) were purchased from Sigma-Aldrich (Seoul, Republic of Korea). Lithium iron (II) phosphate (LiFePO_4_), carbon black (super P) and poly (vinylidene fluoride) (PVDF) were ordered from Alfa Aesar (Seoul, Republic of Korea). CR2032 coin cell kits, aluminum foil, and lithium foil (0.25 mm) were acquired from MTI Corporation (Richmond, CA, USA).

### 2.2. Preparation of Lithium FluoroSulfonyl Oleic Acid (FSOA) Electrolyte

#### 2.2.1. Synthesis of Fluorosulfonyl Isocyanate (FSO_2_NCO)

**Scheme 1:**
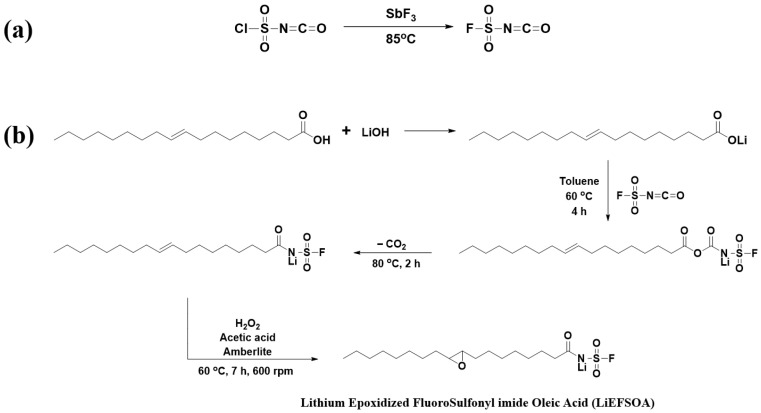
Synthesis of (**a**) fluorosulfonyl isocyanate and (**b**) lithium epoxidized fluorosulfonyl-imide oleic acid (LiEFSOA).

Scheme 1a shows the synthetic route to FSO_2_NCO, which we synthesized by slightly modifying a previously reported method [47]. Antimony trifluoride (100.10 g, 0.56 mol) and chlorosulfonyl isocyanate (240 g, 1.7 mol) were placed in a two-necked round-bottom flask under a nitrogen atmosphere. The reaction temperature was slowly increased to 70 °C and the mixture was stirred for 48 h, followed by Dean–Stark distillation (85 °C) to obtain pure, colorless FSO_2_NCO (yield = 94%).

#### 2.2.2. Synthesis of Lithium Epoxidized Fluorosulfonyl Imide Oleic Acid (LiEFSOA)

Scheme 1b illustrates the overall synthetic route for lithium epoxidized fluorosulfonyl imide oleic acid (LiEFSOA). Oleic acid (20 g, 0.0708 mol) was reacted with LiOH (2.97 g, 0.0708 mol in 200 mL of DI water) in a nitrogen-filled two-necked flask. The mixture was stirred at 0 °C for 1 h and then at 40 °C for 2 h. After filtration, drying at 60 °C for 48 h afforded oleic acid lithium salt (LiOA) as a white solid in 91% yield. LiOA (18 g, 0.0642 mol) was placed in a nitrogen-filled two-necked flask and dispersed in anhydrous toluene (100 mL) with stirring. After cooling the suspension to 0 °C, fluorosulfonyl isocyanate (15.6 g, 0.125 mol) was added dropwise, and the mixture was stirred for 2 h. The reaction was subsequently heated stepwise to 60 °C (4 h) and 80 °C (2 h). Following completion, the solvents and residual isocyanate were removed under reduced pressure, and three successive cycles of addition and evaporation with dried acetonitrile yielded lithium fluorosulfonyl imide oleic acid (LiFSOA) as a viscous dark-red product (25.8 g).

For the epoxidation of the synthesized LiFSOA, we followed previously reported references [48]. Briefly, LiFSOA (10 g, 0.0271 mol), acetic acid (0.182 g, 0.0135 mol), and Amberlite IRC-120H (3 g, 30 wt%) were added to a two-necked flask and stirred under a nitrogen atmosphere. After the mixture was heated to 40 °C, hydrogen peroxide (3.95 g, 0.0406 mol) was added dropwise, and the reaction was maintained at 60 °C with stirring (600 rpm) for 7 h. To terminate the reaction and remove residual hydrogen peroxide, warm deionized water was added and stirred vigorously. The Amberlite was removed by filtration, and the organic layer was washed sequentially with saturated NaHCO_3_, DI water, and brine. Drying over anhydrous MgSO_4_, filtration, and rotary evaporation yielded viscous, bright yellow LiEFSOA in 84% yield.

#### 2.2.3. Preparation of In Situ-Polymerized FSOA Electrolyte

The in situ-polymerized electrolytes were prepared using LiEFSOA, ESO, LiFSI, and EC/DEC solvents. According to polymerization tests, 2 M LiFSI was applied as the lithium salt, after initially dissolving it in EC/DEC (0.2 g, weight ratio 1:1). Subsequently, LiEFSOA and ESO were added in the ratios specified in Table 1 and stirred to obtain a homogeneous solution, which was kept in an Ar-filled glovebox at 50 °C for 3 h. The samples containing 40, 50, and 60 wt% LiEFSOA are denoted as FSOA-1, FSOA-2, and FSOA-3, respectively. A photograph of the in situ-formed FSOA is shown in Figure S1, and additional FSOA and ESO electrolyte compositions are summarized in Table S1.

### 2.3. Fabrication of Coin Cells

#### 2.3.1. Preparation of LFP Cathode

The LFP cathode was fabricated as follows: lithium iron phosphate (LFP), PVDF, and Super P were mixed in a weight ratio of 8:1:1 and dispersed in N-methyl-2-pyrrolidone (NMP) to obtain a homogeneous slurry. The cathode slurry was homogenized using a ball mill and subsequently cast onto Al foil with a doctor blade coater. The electrodes were dried at 100 °C in an oven for 12 h, followed by vacuum drying at 100 °C for another 12 h. The dried LFP cathode was pressed using a roller press (SP-DG200L, Sinopro, Daejeon, Republic of Korea) and then punched into circular electrodes with a diameter of 13 mm in an argon atmosphere.

#### 2.3.2. Cell Assembly and In Situ Polymerization Procedure

Coin cells (CR2032) were assembled individually for each measurement. For ionic conductivity, SS spacers served as both electrodes, with a separator impregnated with stirred FSOA electrolyte placed in between to form a symmetric cell. To determine electrolyte stability via LSV, SS was employed as the working electrode and Li metal as the counter-electrode, with the electrolyte-impregnated separator sandwiched between them. For lithium-ion mobility measurements, symmetric Li|electrolyte|Li cells were assembled using Li metal as both electrodes. Cells were fabricated with an LFP cathode and Li metal anode to evaluate battery performance. All cells were assembled in an Ar-filled glovebox, sealed, and stored at 50 °C for 3 h to allow in situ polymerization.

### 2.4. Methods

#### 2.4.1. Characterization and Measurements

The chemical structures of lithium-substituted monomers and Li-EFSOA were analyzed by FT-IR spectroscopy (Nicolet Summit, Thermo Fisher Scientific, USA) in the range of 4000–500 cm^−1^ at a resolution of 4 cm^−1^. X-ray diffraction (XRD) patterns were recorded using a Rigaku Miniflex diffractometer (Cu Kα radiation, λ = 1.54 Å) to evaluate the crystallinity of the FSOA electrolyte. The interfacial morphology between the polymer electrolyte and the electrode was examined using a table-top scanning electron microscope (SEM, SNE-Alpha, SEC, Republic of Korea) operated at an accelerating voltage of 15 kV. The thermal stability of the FSOA electrolytes was evaluated by thermogravimetric analysis (TGA) using a TGA–N1000 analyzer (Scinco, Gangnam, Republic of Korea) in the temperature range from 25 to 800 °C at a heating rate of 10 °C min^−1^ under an inert N_2_ atmosphere. Differential scanning calorimetry (DSC) was performed to determine the glass transition temperature (*T_g_*) of the electrolytes with a DSC 6000 instrument (PerkinElmer, Tempe, AZ, USA), over the range of −70 to 50 °C at a heating rate of 10 °C min^−1^ under nitrogen flow.

#### 2.4.2. Electrochemical Performance

Electrochemical impedance spectroscopy (EIS) was employed to evaluate the ionic conductivity of FSOAs using an IM6ex analyzer (Zahner–Elektrik, Kronach, Germany). Measurements were conducted from room temperature to 80 °C over a frequency range of 1 MHz to 10 Hz with an AC amplitude of 10 mV. The ionic conductivity of the FSOA was calculated according to

(1) $$\sigma =\frac{l}{RA}$$

where σ (S cm^−1^) is the ionic conductivity; *l* (cm) and *A* (cm^2^) are the thickness and electrode area of the polymer electrolyte, respectively [49]; and *R* (Ω) represents the bulk resistance of the FSOA electrolyte. Meanwhile, the activation energy (E) of the as-prepared FSOAs could be determined by the Arrhenius equation [50,51]:

(2) $$\sigma =Aexp\left(\frac{{-E}_{a}}{kT}\right)$$

where *A* represents the pre-exponential factor, *k* represents the Boltzmann constant, and *T* is the Kelvin temperature.

The lithium-ion transference number (t^+^) of the FSOA electrolyte was determined using chronoamperometry (CA) combined with electrochemical impedance spectroscopy (EIS), recorded both before and after CA. CA was performed at an applied voltage of 10 mV for 25,000 s, while EIS spectra were obtained in the frequency range of 1 MHz to 10 Hz with a perturbation amplitude of 10 mV. The value of t^+^ was calculated according to Equation (3), based on the Bruce–Vincent method,

(3) $$t^{+}=\frac{I_{s}(\Delta V-I_{0}R_{0})}{I_{0}(\Delta V-I_{s}R_{s})}$$

where t^+^ is the lithium-ion transference number; I_0_ and Iₛ are the initial and steady-state currents, respectively; ΔV is the applied DC voltage; and R_0_ and Rₛ are the initial and steady-state interfacial resistances of the polymer electrolyte, respectively.

Linear sweep voltammetry (LSV) of FSOA was carried out over the potential range of −1.5 to 6 V (vs. Li/Li^+^) at a scan rate of 1 mV s^−1^. All CA, LSV, and CV measurements were performed using a Squidstat Plus potentiostat (Admiral Instruments, Tempe, AZ, USA).

Linear sweep voltammetry (LSV) of FSOA was carried out over the potential range of −1.5 to 6 V (vs. Li/Li^+^) at a scan rate of 1 mV s^−1^. All CA, LSV, and CV measurements were performed using a Squidstat Plus potentiostat (Admiral Instruments, Tempe, AZ, USA).

Battery performance tests were conducted by cycling the cells between 2.5 and 4.0 V (vs. Li/Li^+^) at various C-rates and 50 °C using a CT4008T battery tester (Neware, Shenzhen, Guangdong, China).

## 3. Results and Discussion

### 3.1. Structural Characterization of LiEFSOA

**Figure 1:**
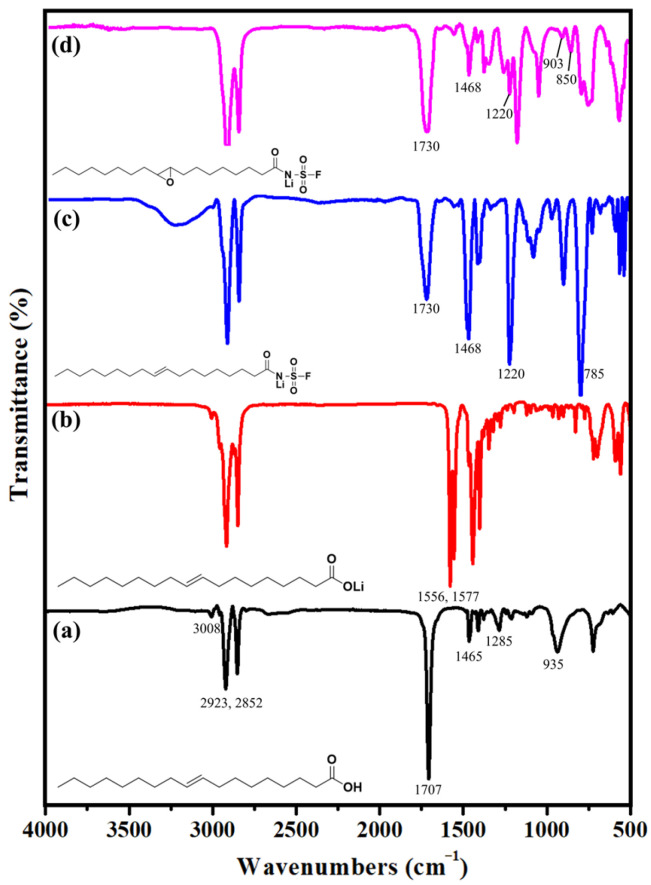
FT-IR spectra of (**a**) oleic acid, (**b**) lithium oleate (LiOA), (**c**) lithium fluorosulfonyl imide oleic acid (LiFSOA), and (**d**) lithium epoxidized fluorosulfonyl imide oleic acid (LiEFSOA).

FTIR spectra were recorded to verify the stepwise synthesis of LiEFSOA, as presented in Figure 1. In the spectrum of oleic acid (a), the characteristic alkene sp^2^ C–H stretching band appeared at 3008 cm^−1^, together with symmetric and asymmetric -CH_2_ stretching peaks at 2852 and 2923 cm^−1^, respectively. A strong absorption band at 1708 cm^−1^ was attributed to -C=O stretching, while those at 1285, 1465, and 935 cm^−1^ corresponded to C-O stretching and O-H vibrations [52,53]. Upon lithiation to form LiOA (b), the disappearance of the O-H peak and the shift in the carbonyl band to 1577 and 1555 cm^−1^ (-COOLi) confirmed the formation of lithium oleate. For LiFSOA (c), new peaks were observed at 1730 cm^−1^ (amide C=O), 1468 cm^−1^ (asymmetric SO_2_ stretching), 1220 cm^−1^ (symmetric SO_2_ stretching), 1073 cm^−1^ (S-N stretching), and 785 cm^−1^ (S-F stretching), demonstrating the successful incorporation of the fluorosulfonyl imide group [47]. In LiEFSOA (d), the disappearance of the alkene band at 3008 cm^−1^ and the emergence of oxirane ring peaks at 850 and 903 cm^−1^ confirmed the epoxidation of LiFSOA. The intermediate reaction pathway was further validated by FTIR data shown in Figure S2, where an anhydride (-CO-O-CO-) intermediate was detected at 60 °C and its subsequent CO_2_ elimination was confirmed after heating at 80 °C [48].

### 3.2. XRD Measurements of FSOAs

**Figure 2:**
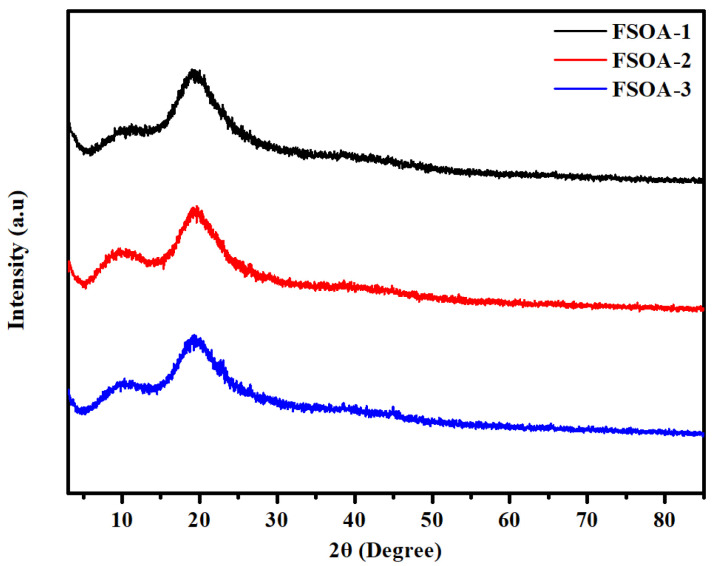
XRD patterns of FSOA electrolytes.

Figure 2 presents the XRD patterns of the FSOA electrolytes. All prepared FSOA samples exhibit pronounced halo features at approximately 2θ ≈ 10° and ≈ 19°. That at 10° corresponds to a d-spacing of about 8.9 Å, and its increasing intensity with higher LiEFOSA content is consistent with a looser packing of polymer chains possibly induced by the long alkyl chains. The broad halo observed near 19° is characteristic of an amorphous polymer coordination environment, supporting the fact that the FSOA electrolytes predominantly possess a non-crystalline structure [54].

### 3.3. Thermal Properties of FSOA Electrolytes

**Figure 3:**
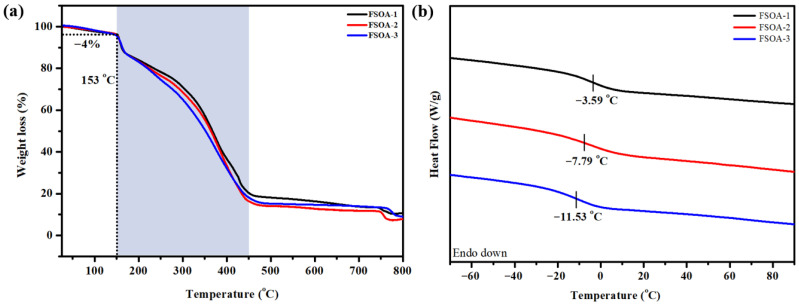
TGA (**a**) and DSC (**b**) curves of FSOA electrolytes.

Thermogravimetric analysis (TGA) and differential scanning calorimetry (DSC) were performed to evaluate the thermal stability and glass transition temperature (*T_g_*) of the FSOA electrolytes, respectively. Figure 3a shows the TGA curves of FSOAs in the temperature range of 25–800 °C. All three electrolytes exhibited an initial mass loss of approximately 4% up to 153 °C, which can be attributed to physical moisture absorption. A sharp weight loss of about 80% was observed between 153 and 450 °C, corresponding to the decomposition of the polymer electrolytes. The extent of weight loss in this region increased with higher LiEFSOA content, likely due to differences in the average molecular weight of the polymers caused by the reduced fraction of oxirane groups [55,56]. These results confirm that the FSOA electrolytes exhibit sufficient thermal stability for operation within the working temperature window of lithium-ion batteries. Figure 3b displays the DSC plots of FSOAs recorded from −70 to 90 °C. The *T_g_* values of FSOA-1, FSOA-2, and FSOA-3 were determined to be −3.59, −7.79, and −11.53 °C, respectively, decreasing with increasing LiEFSOA content, which can be attributed to the long alkyl chains of LiEFSOA imparting additional flexibility within the polymer network. These results are consistent with the XRD pattern described previously. Lower *T_g_* values reflect the enhanced chain flexibility of the polymer electrolytes, thereby promoting higher ionic conductivity and more efficient Li^+^ transport [57,58,59].

### 3.4. Ionic Conductivities and Electrochemical Properties

**Figure 4:**
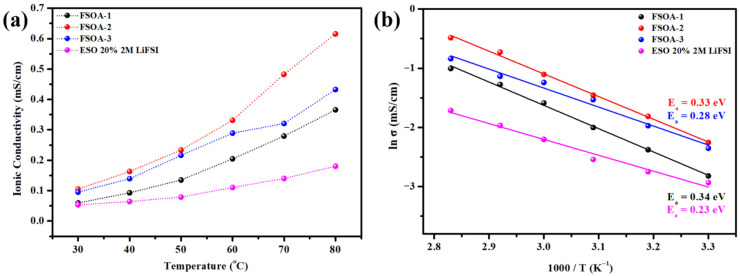
(**a**) Temperature-dependent ionic conductivities of as-prepared FSOAs and ESO electrolyte in the range of 30 to 80 °C. (**b**) Arrhenius plots of the corresponding measured ionic conductivity of FSOAs and ESO electrolyte.

Figure 4a shows the ionic conductivity changes of the prepared FSOA electrolytes and the ESO electrolyte (see Table S1), measured as a control over the temperature range of 30–80 °C. These data were derived from the resistance values shown in Figure S3a–d from the SI. The conductivities of the synthesized electrolytes at 30 °C were 5.33 × 10^−5^, 5.96 × 10^−5^, 9.51 × 10^−5^, and 1.05 × 10^−4^ S cm^−1^, in the order of ESO < FSOA-1 < FSOA-3 < FSOA-2. As the temperature increased, the conductivity of each electrolyte also increased, reaching 1.80 × 10^−4^, 3.66 × 10^−4^, 4.32 × 10^−4^, and 6.15 × 10^−4^ S cm^−1^ at 80 °C in the same order. All FSOA electrolytes exhibited higher ionic conductivities than the ESO-only electrolytes, which can be attributed to the fluorosulfonyl imide-based anions within LiEFSOA, which promote charge delocalization and facilitate lithium-ion transport [60]. Notably, the conductivity of FSOA-2 was higher than those of FSOA-1 and FSOA-3, suggesting that an appropriate balance between the traditional segmental relaxation of the ESO polymer chains and the hopping transport through the fixed anions of LiEFSOA is likely associated with the observed enhancement in ionic conductivity [36,61,62]. Figure 4b shows the Arrhenius plots of each electrolyte, and the calculated activation energies (Eₐ) are 0.34, 0.33, 0.28, and 0.23 eV for FSOA-1, FSOA-2, FSOA-3, and 2M LiFSI with 20% ESO, respectively, which are similar to the activation energies (Eₐ) of typical polymer electrolytes [63,64,65]. The FSOA electrolyte exhibits a higher Eₐ value than the ESO-only electrolyte does. This increase is believed to be due to local confinement effects resulting from the introduction of lithium-ion binding sites within the polymer. FSOA has a relatively high activation energy, but its simultaneous introduction of lithium-ion conductive sites and transport pathways results in a higher overall ionic conductivity than ESO alone [47,66,67]. Additionally, the differences in ionic conductivity according to the ratio of EC/DEC added to the FSOA and ESO electrolytes and the concentration of LiFSI can be seen in Figure S4.

**Figure 5:**
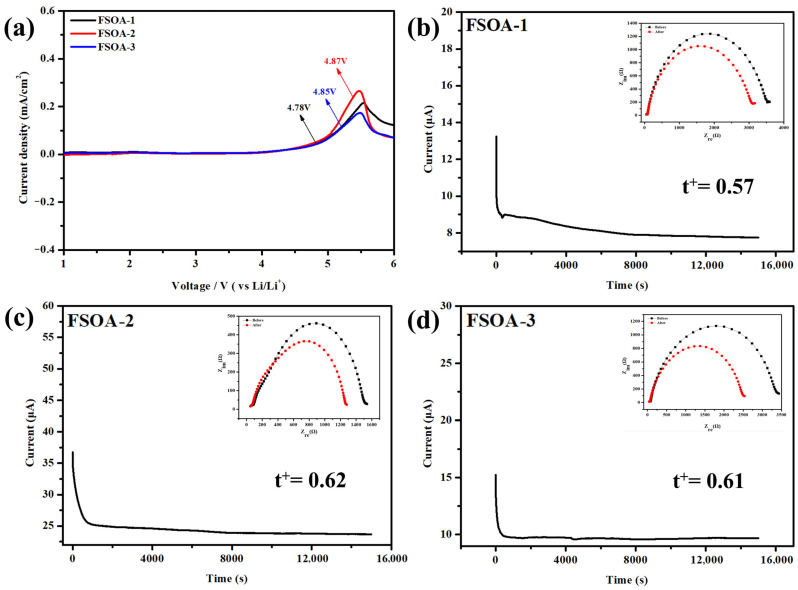
Electrochemical stability and Li^+^ transport properties of FSOA-based electrolytes. (**a**) LSV curves of FSOA-1 to −3, indicating oxidative stability up to 4.8 V vs. Li/Li^+^. (**b**–**d**) Chronoamperometry (CA) and impedance spectra before/after polarization for FSOA-1, FSOA-2, and FSOA-3, respectively.

The electrochemical stability window for FSOA electrolytes is shown in Figure 5a. FSOA-1, FSOA-2, and FSOA-3 started to oxidize at potentials of 4.78, 4.87, and 4.85 V, respectively. Additionally, LSV data for FSOA and ESO electrolytes with different ratios are shown in Figure S5a–c. According to this ESW (electronic stability window), FSOA is more electrochemically stable than LiFSI electrolytes based on PEO (4.0–4.2 V) [68,69] and LiTFSI electrolytes based on PEO (4.2–4.5 V) [70,71]. This suggests that it can be applied not only to LFP electrodes with a redox potential of 3.4 V (vs. Li/Li^+^), but also to batteries using NCM cathodes used in high-voltage batteries. Figure 5b–d present the chronoamperometry (CA) profiles at 10 mV used to determine the lithium-ion transference numbers (TNs, t^+^) of the FSOA electrolytes, together with the electrochemical impedance spectroscopy (EIS) plots recorded before and after CA. The polarization currents stabilized within 15,000 s. Based on Equation (3), TN was 0.57, 0.62, and 0.61 for FSOA-1, FSOA-2, and FSOA-3, respectively. The t^+^ values of FSOA and ESO electrolytes with various compositions are summarized in Figure S6 and Table S2. Among them, FSOA-2 showed the highest lithium-ion transport ability, suggesting that the optimized LiFSI concentration and the free lithium ions in LiEFSOA increased the overall lithium content, while the anionic moieties in LiEFSOA contributed to channel formation that facilitated Li^+^ migration.

Overall, the LiEFSOA-based FSOA electrolyte exhibits enhanced ionic conductivity, a moderately high lithium-ion transference number, and broad electrochemical stability. While each of these parameters is important for evaluating electrolyte performance, assessing the practical value of solid polymer electrolytes requires a balance of these properties rather than a single indicator. To more clearly place this electrolyte system within the context of polymer electrolytes, a comparative summary of the electrochemical properties of solid polymer electrolytes derived from various renewable precursors is presented in Table 2. Polymer electrolytes derived from renewable precursors reported in the literature achieve a high lithium-ion TN but suffer from low ionic conductivity and poor oxidation stability.

The LiEFSOA-based FSOA electrolyte developed in this study achieves a good balance between ionic conductivity (ca. 10^−4^ S cm^−1^), a moderately high lithium-ion transference number (t^+^ ≈ 0.62), and a wide electrochemical stability (≈4.8 V vs. Li^+^/Li). This balanced performance differentiates this system from previously reported bio-derived polymer electrolytes and highlights the effectiveness of the proposed hybrid transport strategy in mitigating the inherent tradeoff between ionic conductivity and lithium-ion selectivity.

### 3.5. Electrochemical Performance of FSOA-Based Cell

**Figure 6:**
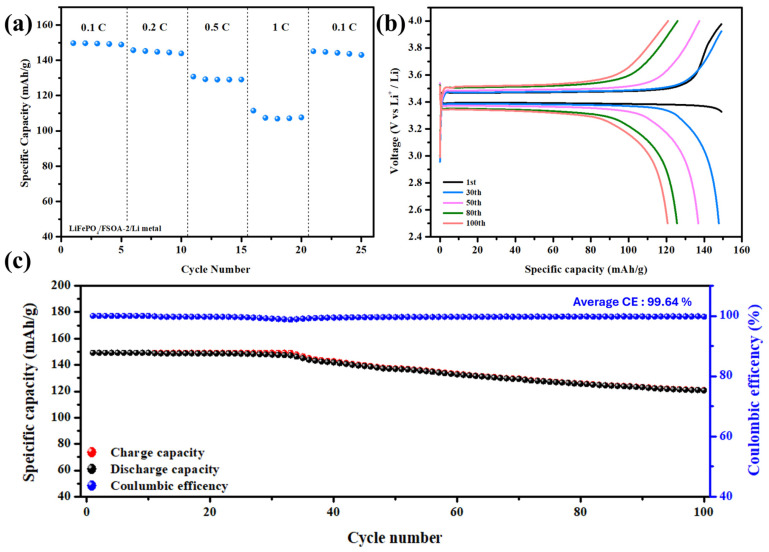
(**a**) Rate performance of the LiFePO_4_/FSOA-2/Li metal cell measured at different C-rates. (**b**) Charge–discharge voltage profiles of the LiFePO_4_/FSOA-2/Li metal cell at different cycle numbers. (**c**) Cycling performance and coulombic efficiency of the LiFePO_4_/FSOA-2/Li metal cell at 0.1 C. The red and black represent the charge and discharge capacities, respectively, and the blue represents the coulombic efficiency.

While the comparison in Table 2 demonstrates the electrochemical performance of this electrolyte at the material level, many reported solid polymer electrolytes lack validation in practical battery configurations. To further demonstrate the practical applicability of the LiEFSOA-based FSOA electrolyte, the charge–discharge performance of the FSOA electrolytes was evaluated using LiFePO4/FSOA-2/Li metal coin cells within a voltage range of 2.5–4.0 V. Figure 6a shows the discharge capacities measured at various C-rates (0.1–1 C). Galvanostatic cycling was carried out for five cycles at each rate of 0.1, 0.2, 0.5, and 1 C, yielding average discharge capacities of 149.36, 144.80, 129.41, and 108.11 mAh g^−1^, respectively. As the current density increased, a gradual decrease in discharge capacity was observed. When the current rate was subsequently returned to 0.1 C, the average discharge capacity recovered to 144.13 mAh g^−1^, corresponding to 96.5% of the initial capacity, indicating good reversibility of the FSOA-2 electrolyte under cycling conditions.

Figure 6b, c show the charge–discharge profiles of the LiFePO_4_/FSOA-2/Li metal cell. The FSOA-2 electrolyte delivered an initial discharge capacity of 149.09 mAh g^−1^ at 0.1 C. This discharge capacity was maintained up to approximately 35 cycles and then gradually decreased with further cycling, reaching 120.9 mAh g^−1^ after 100 cycles, corresponding to a capacity retention of 81.09%. The average coulombic efficiency during cycling was 99.62%, indicating highly reversible electrochemical behavior. As shown in Figure 5b, although the FSOA-2 electrolyte exhibits stable coulombic efficiency during cycling, the gradual capacity decay and increased voltage hysteresis observed after prolonged cycling indicate the inherent limitations of the present system. One possible cause of this behavior is the presence of residual unreacted monomers and oligomers that may undergo further polymerization or side reactions under repeated electrochemical cycling, potentially leading to chemical instability and heterogeneous ion-transport pathways [76]. In addition, partial degradation of the polymer matrix or lithium salt during long-term cycling may contribute to the progressive buildup of bulk and interfacial resistance, as reflected by the increased voltage hysteresis during cycles [77]. These effects suggest that, while the current electrolyte design enables practical cell operation, further optimization of the polymerization completeness and interfacial stability will be required to improve long-term cycling durability.

### 3.6. Structure–Property Relationship in the FSOA Electrolyte and In Situ Methods

**Figure 7:**
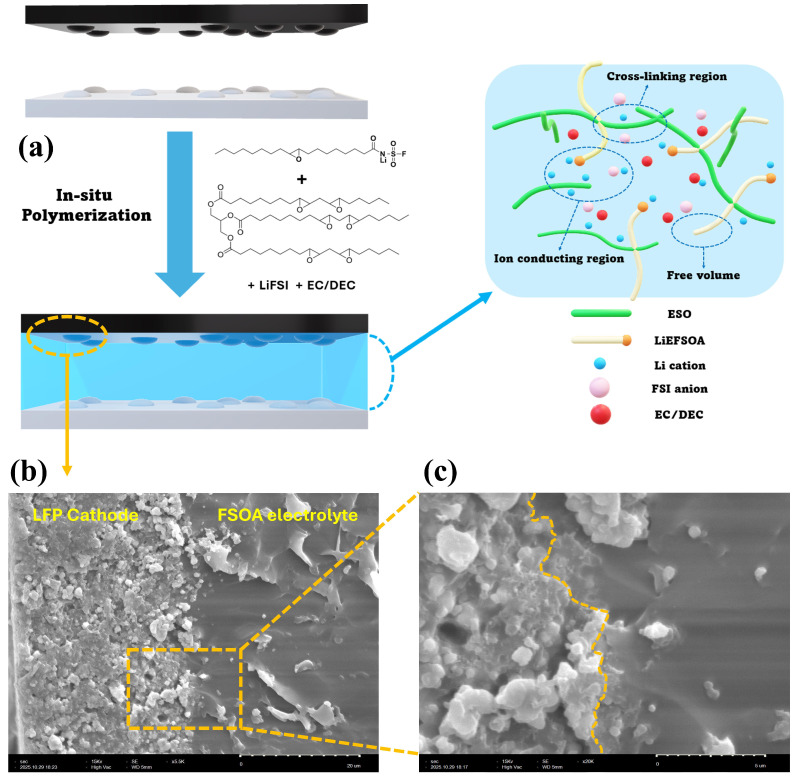
(**a**) Conceptual illustration of the in situ formation of the FSOA polymer electrolyte, highlighting the cross-linked network, ion-conducting regions, and internal free volume; (**b**) cross-sectional SEM image of the LiFePO_4_ (LFP) cathode and the FSOA polymer electrolyte after in situ polymerization; and (**c**) magnified cross-sectional SEM image of the LFP/FSOA electrolyte interface.

These results can be explained by the schematic diagram shown in Figure 7a. The in situ-polymerized polymer electrolyte, consisting of the ion-conducting monomer LiEFSOA, the cross-linking network ESO, and the lithium salt LiFSI, provides mechanical strength through the cross-linked matrix while facilitating efficient Li^+^ ion transport through the ion-conducting domains. Furthermore, the long hydrocarbon chains of LiEFSOA are expected to contribute to increased internal free volume and suppressed crystallinity, consistent with the predominantly amorphous nature observed in XRD. Additionally, Figure 7b,c show cross-sectional SEM images of the LFP cathode and the FSOA electrolyte. The in situ polymerization process, which involves sufficient impregnation of the electrolyte precursor into the electrode followed by polymerization, confirms the formation of a close interface between the electrode and the electrolyte. The smooth interfacial contact reduces interfacial resistance, thereby further improving ionic conductivity. Overall, this integrated structural strategy synergistically enhances the electrochemical performance of FSOA-based electrolytes.

## 4. Conclusions

In summary, we developed an in situ-polymerized, oleic-acid-derived hybrid polymer electrolyte that combines the advantages of single-ion selectivity and dual-ion conductivity. The FSOA-based electrolyte exhibited excellent thermal stability, as evidenced by a decomposition temperature above 153 °C and a low glass transition temperature (*T_g_*), and showed a wide electrochemical stability window up to 4.87 V versus Li^+^/Li. By introducing an in situ polymerization strategy together with a hybrid electrolyte design, the electrolyte achieved an enhanced Li^+^ transference number (t^+^) of 0.62 and a stable ionic conductivity of 1.05 × 10^−4^ S cm^−1^ at 30 °C, indicating both improved cation-selective transport and reliable ion conduction. When applied to LiFePO_4_/FSOA-2/Li metal cells, the FSOA-2 electrolyte delivered an initial discharge capacity of 149.09 mAh g^−1^ and maintained 81.09% of its capacity after 100 cycles, along with a high average coulombic efficiency of 99.62%. While the present system demonstrates feasible electrochemical performance under the investigated conditions, further improvement in electrolyte performance at ambient and lower temperatures will be required, and such optimization remains an important subject for future studies. These results demonstrate that the combination of bio-based materials, hybrid ion conduction, and in situ interfacial formation provides a promising platform for sustainable polymer electrolytes for lithium-ion batteries.

## Figures and Tables

**Table 1:** Composition and ratio of FSOA.

Electrolyte Name	Weight of LiEFSOA (g)	Weight of ESO (g)	Ratio	Weight of EC/DEC (g)	Weight of LiFSI (g)
FSOA-1	0.32	0.48	4:6	0.2	0.374
FSOA-2	0.4	0.4	5:5	0.2	0.374
FSOA-3	0.48	0.32	6:4	0.2	0.374

**Table 2:** Electrochemical properties of solid polymer electrolytes derived from various renewable precursors.

Electrolyte System	Renewable Precursor	Salt	Electrochemical Properties				Tg (°C)	Ref.
			σ (s/cm)	I-TN	Stability (V)	E_a_ (eV)		
FSOA-2	Oleic acid	LiFSI	1.05 × 10^−4^	0.62	4.87	0.33	−7.79	This work
PU	Falm oil	LiCF_3_SO_3_	1.6 × 10^−5^	-	-	-	-	[72]
PU-LiClO_4_	Jatropha oil	LiClO_4_	1.29 × 10^−4^	0.83	4.8	-	-	[73]
PU-LiI	Castor oil	LiI	1.78 × 10^−6^	0.99	2.0	0.13	−27.5	[74]
poly(1)	D-xylose 10-undecenoic acid	LiTFSI	1.0 × 10^−5^	0.84	3.88	-	-	[75]

## Data Availability

The original contributions presented in this study are included in the article/Supplementary Material. Further inquiries can be directed to the corresponding author.

## Supplementary Materials

The following supporting information can be downloaded at https://www.mdpi.com/article/10.3390/polym18060773/s1. Table S1. The manufacturing ratio of FSOA and ESO (reference) polymer electrolyte. Table S2. Lithium-ion transference numbers (t^+^ₗᵢ) of FSOA- and ESO-based polymer electrolytes. Figure S1. Polymerization test of FSOA-1, FSOA-2, FSOA-3 and FSOA-4. Figure S2. FTIR spectra of LiFSOA obtained after reaction at 60 °C for 4 h (black) and after subsequent heating at 80 °C for 2 h (red). At 60 °C, characteristic absorptions at 1810 cm^−1^ and 1037 cm^−1^ indicate the presence of an anhydride-related intermediate (O=C–O–C=O). Upon further heating to 80 °C, these features disappeared, accompanied by the emergence of a strong amide C=O absorption at 1730 cm^−1^, confirming CO_2_ elimination and successful formation of LiFSOA. Figure S3. EIS plots of (a) FSOA-1, (b) FSOA-2, (c) FSOA-3 and (d)ESO 20% 2M electrolytes. Figure S4. ionic conductivity vs. temperature plots of varied concentrated LiFSI and EC/DEC in FSOA (5:5 wt ratio) and ESO electrolytes. Figure S5. Linear sweep voltammetry (LSV) curves of (a) FSOA-5, FSOA-6, and FSOA-7, (b) FSOA-8, FSOA-9, and FSOA-10, and (c) ESO-based electrolytes with different salt concentrations, measured at room temperature with SS/electrolyte/Li cells. Figure S6. Chronoamperometry (CA) and impedance spectra before/after polarization plot of FSOAs and ESO-based electrolytes with different salt concentrations.

## References

[1] Makeyaw A., Dame T., Getu M., Goytom D. (2025). Targeted Advances in Lithium-ion Batteries: A Critical Review of Synergetic Improvements in Energy Density, Life Cycle, and Safety. Am. J. Quantum Chem. Mol. Spectrosc..

[2] Ngoy K.R., Lukong V.T., Yoro K.O., Makambo J.B., Chukwuati N.C., Ibegbulam C., Eterigho-Ikelegbe O., Ukoba K., Jen T.-C. (2025). Lithium-ion batteries and the future of sustainable energy: A comprehensive review. Renew. Sustain. Energy Rev..

[3] Yang H., Wu N. (2022). Ionic conductivity and ion transport mechanisms of solid-state lithium-ion battery electrolytes: A review. Energy Sci. Eng..

[4] Lin J., Xu W., Dong W., Tan J., Wang R., Zhang Z., Liu Q., Yin G., Zhu C., Xu J. (2025). High-Voltage-Resistant Highly Stable Solid Polymer Electrolyte via In Situ Integrated Construction with Fast Ion Migration. ACS Appl. Mater. Interfaces.

[5] Ngo H.P.K., Shao Y., Bertaux T., Nguyen T.K.L., Solier J., Planes E., Judeinstein P., Alloin F., Sanchez J.-Y., Iojoiu C. (2025). Single-ion conducting polymer electrolyte with excellent interfacial stability toward the lithium metal. ACS Appl. Energy Mater..

[6] Fan X., Wang C. (2021). High-voltage liquid electrolytes for Li batteries: Progress and perspectives. Chem. Soc. Rev..

[7] Feng J., Wang L., Chen Y., Wang P., Zhang H., He X. (2021). PEO based polymer-ceramic hybrid solid electrolytes: A review. Nano Converg..

[8] Hu X., Wang Y., Feng X., Wang L., Ouyang M., Zhang Q. (2025). Thermal stability of ionic liquids for lithium-ion batteries: A review. Renew. Sustain. Energy Rev..

[9] Wang J., Zhang C., Zhang Y., Xue Z. (2022). Advances in host selection and interface regulation of polymer electrolytes. J. Polym. Sci..

[10] Zhang M., Wang L., Xu H., Song Y., He X. (2023). Polyimides as promising materials for lithium-ion batteries: A review. Nano-Micro Lett..

[11] Abraham K., Jiang Z., Carroll B. (1997). Highly conductive PEO-like polymer electrolytes. Chem. Mater..

[12] Zhang X., Liu T., Zhang S., Huang X., Xu B., Lin Y., Xu B., Li L., Nan C.-W., Shen Y. (2017). Synergistic coupling between Li6. 75La3Zr1. 75Ta0. 25O12 and poly (vinylidene fluoride) induces high ionic conductivity, mechanical strength, and thermal stability of solid composite electrolytes. J. Am. Chem. Soc..

[13] Song Y., Su M., Xiang H., Kang J., Yu W., Peng Z., Wang H., Cheng B., Deng N., Kang W. (2025). PEO-Based Solid-State Polymer Electrolytes for Wide-Temperature Solid-State Lithium Metal Batteries. Small.

[14] Tan J., Guo L., Hu J., Liu S. (2024). Recent advances in poly (ethylene oxide)-based solid-state electrolytes for lithium-ion batteries. J. Phys. Chem. C.

[15] Jin L., Jang G., Lim H., Zhang W., Park S., Jeon M., Jang H., Kim W. (2022). Improving the ionic conductivity of PEGDMA-based polymer electrolytes by reducing the interfacial resistance for LIBs. Polymers.

[16] Sundaramahalingam K., Vanitha D., Nallamuthu N., Manikandan A., Muthuvinayagam M. (2019). Electrical properties of lithium bromide poly ethylene oxide/poly vinyl pyrrolidone polymer blend elctrolyte. Phys. B Condens. Matter.

[17] Arya A., Sharma A. (2017). Polymer electrolytes for lithium ion batteries: A critical study. Ionics.

[18] Ngai K.S., Ramesh S., Ramesh K., Juan J.C. (2016). A review of polymer electrolytes: Fundamental, approaches and applications. Ionics.

[19] Zhang Z., Cui K., Zhang J.m., Xu Y., Xu F. (2025). Cellulose-Based Solid-State Electrolytes for Solid-State Lithium Metal Batteries: Advances in Functionalization Strategies and Interfacial Engineering. Adv. Energy Mater..

[20] Tordi P., Montes-García V., Tamayo A., Bonini M., Samorì P., Ciesielski A. (2025). Ionically Tunable Gel Electrolytes Based on Gelatin-Alginate Biopolymers for High-Performance Supercapacitors. Small.

[21] Huo X., Shi X., An L. (2025). Towards Bio-derived Electrolytes for Sustainable Redox Flow Batteries. Chem. Res. Chin. Univ..

[22] Huang J., Wang S., Chen J., Chen C., Lizundia E. (2025). Environmental Sustainability of Natural Biopolymer-Based Electrolytes for Lithium Ion Battery Applications. Adv. Mater..

[23] Fu Y., Yang L., Zhang M., Lin Z., Shen Z. (2022). Recent advances in cellulose-based polymer electrolytes. Cellulose.

[24] Yahya M., Arof A. (2003). Effect of oleic acid plasticizer on chitosan–lithium acetate solid polymer electrolytes. Eur. Polym. J..

[25] Chai M., Isa M. (2016). Novel proton conducting solid bio-polymer electrolytes based on carboxymethyl cellulose doped with oleic acid and plasticized with glycerol. Sci. Rep..

[26] Zhang W., Bae W., Jin L., Park S., Jeon M., Kim W., Jang H. (2023). Cross-Linked Gel Polymer Electrolyte Based on Multiple Epoxy Groups Enabling Conductivity and High Performance of Li-Ion Batteries. Gels.

[27] Rösch J., Mülhaupt R. (1993). Polymers from renewable resoureces: Polyester resins and blends based upon anhydride-cured epoxidized soybean oil. Polym. Bull..

[28] Ma X., Yu J., Zou X., Hu Y., Yang M., Zhang F., Yan F. (2023). Single additive to regulate lithium-ion solvation structure in carbonate electrolytes for high-performance lithium-metal batteries. Cell Rep. Phys. Sci..

[29] Wang Q., Zhao C., Wang J., Yao Z., Wang S., Kumar S.G.H., Ganapathy S., Eustace S., Bai X., Li B. (2023). High entropy liquid electrolytes for lithium batteries. Nat. Commun..

[30] Sun S.-Y., Zhang X.-Q., Wang Y.-N., Li J.-L., Zheng Z., Huang J.-Q. (2024). Understanding the transport mechanism of lithium ions in solid-electrolyte interphase in lithium metal batteries with liquid electrolytes. Mater. Today.

[31] Chang R., Liu Y., Zhang Y., Shi Y., Tang J., Xu Z.L., Zhou X., Yang J. (2025). Weakening Lithium-Ion Coordination in Poly (Ethylene Oxide)-Based Solid Polymer Electrolytes for High Performance Solid-State Batteries. Adv. Energy Mater..

[32] Bresser D., Lyonnard S., Iojoiu C., Picard L., Passerini S. (2019). Decoupling segmental relaxation and ionic conductivity for lithium-ion polymer electrolytes. Mol. Syst. Des. Eng..

[33] Torell L., Schantz S. (1991). Structural relaxation and chain flexibility in polymeric ion conductors. J. Non-Cryst. Solids.

[34] Ratner M.A., Johansson P., Shriver D.F. (2000). Polymer electrolytes: Ionic transport mechanisms and relaxation coupling. MRS Bull..

[35] Ock J.-y., Lehmann M., Li C., Wang Y., Meyer H.M., Sokolov A.P., Fu Z., Chen X.C. (2025). A single-ion-conducting polymer and high-entropy Li-garnet composite electrolyte with simultaneous enhancement in ion transport and mechanical properties. J. Mater. Chem. A.

[36] Gao J., Wang C., Han D.-W., Shin D.-M. (2021). Single-ion conducting polymer electrolytes as a key jigsaw piece for next-generation battery applications. Chem. Sci..

[37] Gan H., Li S., Zhang Y., Yu L., Wang J., Xue Z. (2021). Mechanically strong and electrochemically stable single-ion conducting polymer electrolytes constructed from hydrogen bonding. Langmuir.

[38] Zhang Y., Yu J., Shi H., Wang S., Lv Y., Zhang Y., Yuan Q., Liang J., Gao T., Wei R. (2025). Fiber-reinforced ultrathin solid polymer electrolyte for solid-state lithium-metal batteries. Adv. Funct. Mater..

[39] Chae W., Kim B., Ryoo W.S., Earmme T. (2023). A brief review of gel polymer electrolytes using in situ polymerization for lithium-ion polymer batteries. Polymers.

[40] Liu T., Zhang J., Han W., Zhang J., Ding G., Dong S., Cui G. (2020). In situ polymerization for integration and interfacial protection towards solid state lithium batteries. J. Electrochem. Soc..

[41] Lee Y.-W., Yoo J.-H., Kim D.-W. High Performance Lithium-Ion Polymer Cells Assembled Using In-Situ Chemical Cross-Linking Without a Free Radical Initiator. Proceedings of the ECS Meeting Abstracts.

[42] Jin S., Shan X., Tian J., Li Y., Ding H., Advincula R., Tian M., Cao P.F. (2025). High-Entropy Gel Polymer Electrolyte for Wide-Temperature Operatable Li-Metal Batteries. Adv. Funct. Mater..

[43] Zhang W., Yoon S., Jin L., Lim H., Jeon M., Jang H., Ahmed F., Kim W. (2022). Lithium salt catalyzed ring-opening polymerized solid-state electrolyte with comparable ionic conductivity and better interface compatibility for Li-ion batteries. Membranes.

[44] Ma Y., Sun Q., Wang S., Zhou Y., Song D., Zhang H., Shi X., Zhang L. (2022). Li salt initiated in-situ polymerized solid polymer electrolyte: New insights via in-situ electrochemical impedance spectroscopy. Chem. Eng. J..

[45] Bartels J., Wang J.-H.H., Chen Q., Runt J., Colby R.H. (2016). Segmental dynamics of ethylene oxide-containing polymers with diverse backbone chemistries. Macromolecules.

[46] Vijayakumar V., Anothumakkool B., Kurungot S., Winter M., Nair J.R. (2021). In situ polymerization process: An essential design tool for lithium polymer batteries. Energy Environ. Sci..

[47] Ahmed F., Choi I., Ryu T., Yoon S., Rahman M.M., Zhang W., Jang H., Kim W. (2020). Highly conductive divalent fluorosulfonyl imide based electrolytes improving Li-ion battery performance: Additive potentiating electrolytes action. J. Power Sources.

[48] Abolins A., Kirpluks M., Vanags E., Fridrihsone A., Cabulis U. (2020). Tall Oil Fatty Acid Epoxidation Using Homogenous and Heterogeneous Phase Catalysts. J. Polym. Environ..

[49] Ramya C., Selvasekarapandian S., Hirankumar G., Savitha T., Angelo P. (2008). Investigation on dielectric relaxations of PVP–NH4SCN polymer electrolyte. J. Non-Cryst. Solids.

[50] Croce F., Focarete M.L., Hassoun J., Meschini I., Scrosati B. (2011). A safe, high-rate and high-energy polymer lithium-ion battery based on gelled membranes prepared by electrospinning. Energy Environ. Sci..

[51] Zhang Y., Feng W., Zhen Y., Zhao P., Wang X., Li L. (2022). Effects of lithium salts on PEO-based solid polymer electrolytes and their all-solid-state lithium-ion batteries. Ionics.

[52] Lee S.-Y., Harris M.T. (2006). Surface modification of magnetic nanoparticles capped by oleic acids: Characterization and colloidal stability in polar solvents. J. Colloid Interface Sci..

[53] Ibarra J., Melendres J., Almada M., Burboa M.G., Taboada P., Juárez J., Valdez M.A. (2015). Synthesis and characterization of magnetite/PLGA/chitosan nanoparticles. Mater. Res. Express.

[54] Bakar R., Darvishi S., Aydemir U., Yahsi U., Tav C., Menceloglu Y.Z., Senses E. (2023). Decoding polymer architecture effect on ion clustering, chain dynamics, and ionic conductivity in polymer electrolytes. ACS Appl. Energy Mater..

[55] Montoya-Ospina M.C., Verhoogt H., Ordner M., Tan X., Osswald T.A. (2022). Effect of cross-linking on the mechanical properties, degree of crystallinity and thermal stability of polyethylene vitrimers. Polym. Eng. Sci..

[56] Krongauz V.V. (2010). Crosslink density dependence of polymer degradation kinetics: Photocrosslinked acrylates. Thermochim. Acta.

[57] Agapov A.L., Sokolov A.P. (2011). Decoupling ionic conductivity from structural relaxation: A way to solid polymer electrolytes?. Macromolecules.

[58] Wang Y., Fan F., Agapov A.L., Saito T., Yang J., Yu X., Hong K., Mays J., Sokolov A.P. (2014). Examination of the fundamental relation between ionic transport and segmental relaxation in polymer electrolytes. Polymer.

[59] Imrie C.T., Ingram M.D. (2001). Decoupled ion transport in mesomorphic polymer electrolyte glasses. Electrochim. Acta.

[60] Ahmed F., Choi I., Rahman M.M., Jang H., Ryu T., Yoon S., Jin L., Jin Y., Kim W. (2019). Remarkable conductivity of a self-healing single-ion conducting polymer electrolyte, poly (ethylene-co-acrylic lithium (fluoro sulfonyl) imide), for all-solid-state Li-ion batteries. ACS Appl. Mater. Interfaces.

[61] Brooks D.J., Merinov B.V., Goddard W.A., Kozinsky B., Mailoa J. (2018). Atomistic description of ionic diffusion in PEO–LiTFSI: Effect of temperature, molecular weight, and ionic concentration. Macromolecules.

[62] Zhang M., Yu S., Mai Y., Zhang S., Zhou Y. (2019). A single-ion conducting hyperbranched polymer as a high performance solid-state electrolyte for lithium ion batteries. Chem. Commun..

[63] Reddy M.J., Sreekanth T., Rao U.S. (1999). Study of the plasticizer effect on a (PEO+ NaYF4) polymer electrolyte and its use in an electrochemical cell. Solid State Ion..

[64] Li C., Xue P., Chen L., Liu J., Wang Z. (2022). Reducing the crystallinity of PEO-based composite electrolyte for high performance lithium batteries. Compos. Part B Eng..

[65] Chandrasekaran R., Sathiyamoorthi R., Selladurai S. (2009). Role of composite MnO2 cathode on electrochemical cells based on polymer electrolyte (PEO/NaClO_3_). Ionics.

[66] Li Z., Fu J., Zhou X., Gui S., Wei L., Yang H., Li H., Guo X. (2023). Ionic conduction in polymer-based solid electrolytes. Adv. Sci..

[67] Imbrogno J., Maruyama K., Rivers F., Baltzegar J.R., Zhang Z., Meyer P.W., Ganesan V., Aoshima S., Lynd N.A. (2021). Relationship between ionic conductivity, glass transition temperature, and dielectric constant in poly (vinyl ether) lithium electrolytes. ACS Macro Lett..

[68] Nie K., Wang X., Qiu J., Wang Y., Yang Q., Xu J., Yu X., Li H., Huang X., Chen L. (2020). Increasing poly (ethylene oxide) stability to 4.5 V by surface coating of the cathode. ACS Energy Lett..

[69] Qiu J., Liu X., Chen R., Li Q., Wang Y., Chen P., Gan L., Lee S.J., Nordlund D., Liu Y. (2020). Enabling stable cycling of 4.2 V high-voltage all-solid-state batteries with PEO-based solid electrolyte. Adv. Funct. Mater..

[70] Yang X., Jiang M., Gao X., Bao D., Sun Q., Holmes N., Duan H., Mukherjee S., Adair K., Zhao C. (2020). Determining the limiting factor of the electrochemical stability window for PEO-based solid polymer electrolytes: Main chain or terminal–OH group?. Energy Environ. Sci..

[71] Du Z., Chen X.C., Sahore R., Wu X., Li J., Dudney N.J. (2021). Effects of plasticizer content and ceramic addition on electrochemical properties of cross-linked polymer electrolyte. J. Electrochem. Soc..

[72] Daud F.N., Ahmad A., Haji Badri K. (2014). An Investigation on the Properties of Palm-Based Polyurethane Solid Polymer Electrolyte. Int. J. Polym. Sci..

[73] Mustapa S.R., Aung M.M., Ahmad A., Mansor A., TianKhoon L. (2016). Preparation and characterization of Jatropha oil-based Polyurethane as non-aqueous solid polymer electrolyte for electrochemical devices. Electrochim. Acta.

[74] Ibrahim S., Ahmad A., Mohamed N.S. (2015). Characterization of novel castor oil-based polyurethane polymer electrolytes. Polymers.

[75] Oshinowo M., Runge J.R., Piccini M., Marken F., Buchard A. (2022). Crosslinked xylose-based polyester as a bio-derived and degradable solid polymer electrolyte for Li+-ion conduction. J. Mater. Chem. A.

[76] Choi M.S., Kang S.G., Choi J., Ko J., Park J.H. (2025). Residual Monomer-Induced Side Reactions in Gel Polymer Electrolytes: Unveiled High-Ni Cathode Failure in Lithium Batteries. Angew. Chem..

[77] Ock J.-y., Kalnaus S., Zachman M.J., Ullman A.M., Owensby K.D., Long O., Chen X.C., Sahore R. (2026). Decoupling the capacity fade contributions in polymer electrolyte-based high-voltage solid-state batteries. J. Mater. Chem. A.

