# Supplementary material for: A Hybrid Oleic-Acid-Derived Polymer Electrolyte Integrating Single- and Dual-Ion Conducting Systems for Lithium-Ion Batteries
Source: Polymers (Basel). 2026 Mar 23;18(6):773. doi: 10.3390/polym18060773 (PMC13030067; doi:10.3390/polym18060773)
Supplement: Supplementary file 1 [file polymers-18-00773-s001.zip › polymers-4180778-supplementary.pdf]

Supplementary information

A Hybrid Oleic-Acid-Derived Polymer Electrolyte Integrati ng Single- and Dual-Ion Conducting Systems for Lithium-Ion Batteries

Wansu Bae, Sutradhar Sabuj Chandra, Doyul Lee, Donghoon Kang, Hyewon Na, Jiye Lee and Hohyoun Jang *

Department of Energy Material Science, Konkuk University, 268 Chungwon-daero, Chungju-si 27478, Republic of Korea; gelp621@naver.com (W.B.); chandra@kku.ac.kr (S.S.C.); b6769@naver.com (D.L.); rkdehdgns28@naver.com (D.K.); gpdnjs3z3@naver.com (H.N.); wldp9902@naver.com (J.L.)

* Correspondence: 200417450@kku.ac.kr

Table S1. The manufacturing ratio of FSOA and ESO (reference) polymer electrolyte.

Electrolyte name	Weight of LiEFSOA (g)	Weight of ESO (g)	Ratio	Weight of EC/DEC (g)	Weight of LiFSI (g)	Polymerization
FSOA-1	0.32	0.48	4:6	0.2	0.374	O
FSOA-2	0.4	0.4	5:5	0.2	0.374	O
FSOA-3	0.48	0.32	6:4	0.2	0.374	O
FSOA-4	0.56	0.24	7:3	0.2	0.374	X
FSOA-5	0.32	0.48	4:6	0.2	0.187	O
FSOA-6	0.4	0.4	5:5	0.2	0.187	O
FSOA-7	0.48	0.32	6:4	0.2	0.187	O
FSOA-8	0.36	0.54	4:6	0.1	0.187	O
FSOA-9	0.45	0.45	5:5	0.1	0.187	O
FSOA-10	0.54	0.36	6:4	0.1	0.187	O
ESO 10% 1M		0.9		0.1	0.187	O
ESO 20% 1M		0.8		0.2	0.187	O
ESO 20% 2M		0.8		0.2	0.374	O

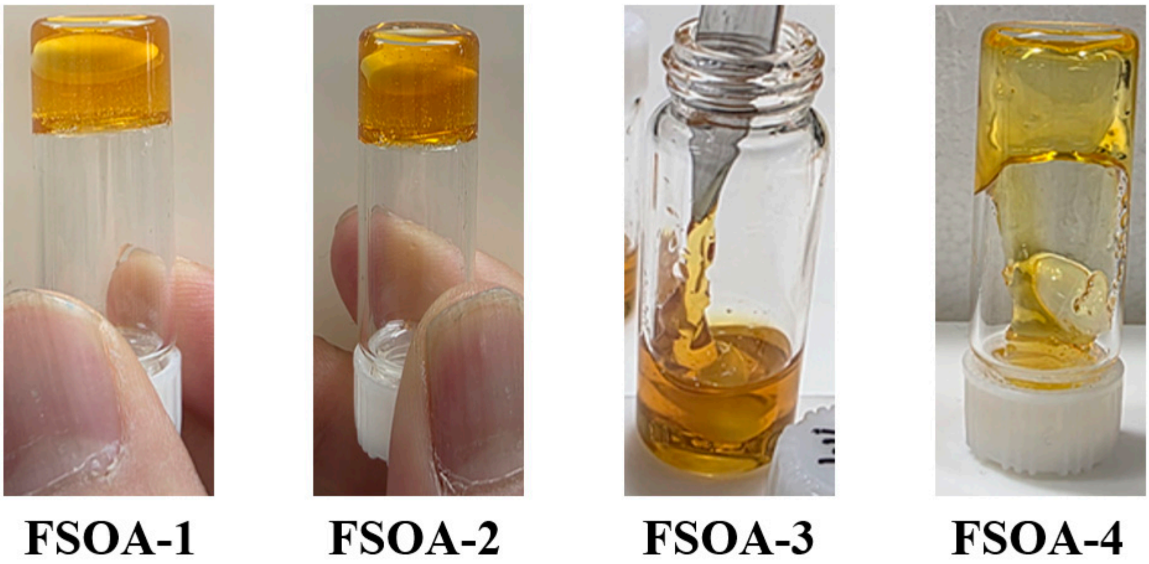

Figure S1. Polymerization test of FSOA-1, FSOA-2, FSOA-3 and FSOA-4

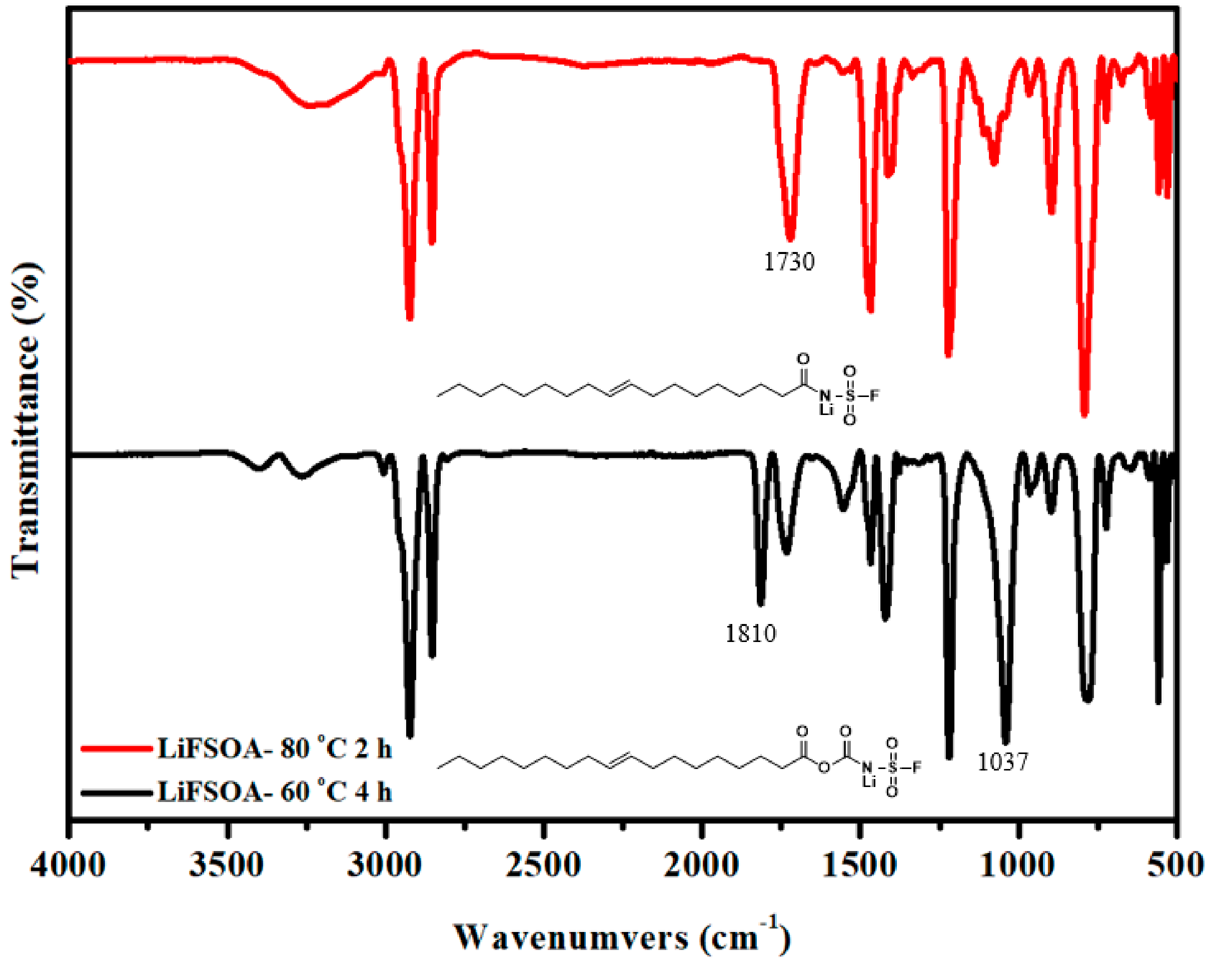

Figure S2. FTIR spectra of LiFSOA obtained after reaction at 60 °C for 4 h (black) and after subsequent heating at 80 °C for 2 h (red). At 60 °C, characteristic absorptions at 1810 cm^{-1} and 1037 cm^{-1} indicate the presence of an anhydride-related intermediate (O=C–O–C=O). Upon further heating to 80 °C, these features disappeared, accompanied by the emergence of a strong amide C=O absorption at 1730 cm^{-1}, confirming CO_2 elimination and successful formation of LiFSOA.

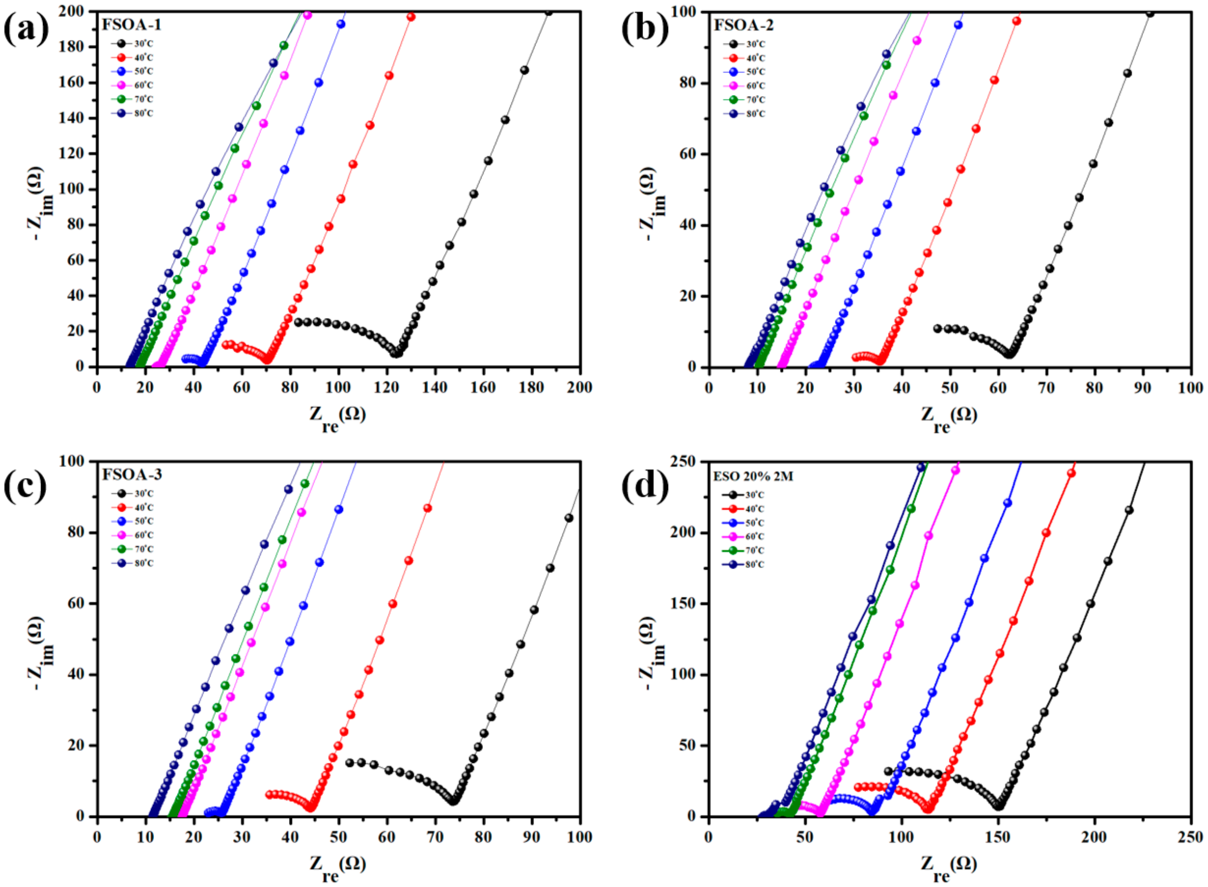

Figure S3. EIS plots of (a) FSOA-1, (b) FSOA-2, (c) FSOA-3 and (d)ESO 20% 2M electrolytes.

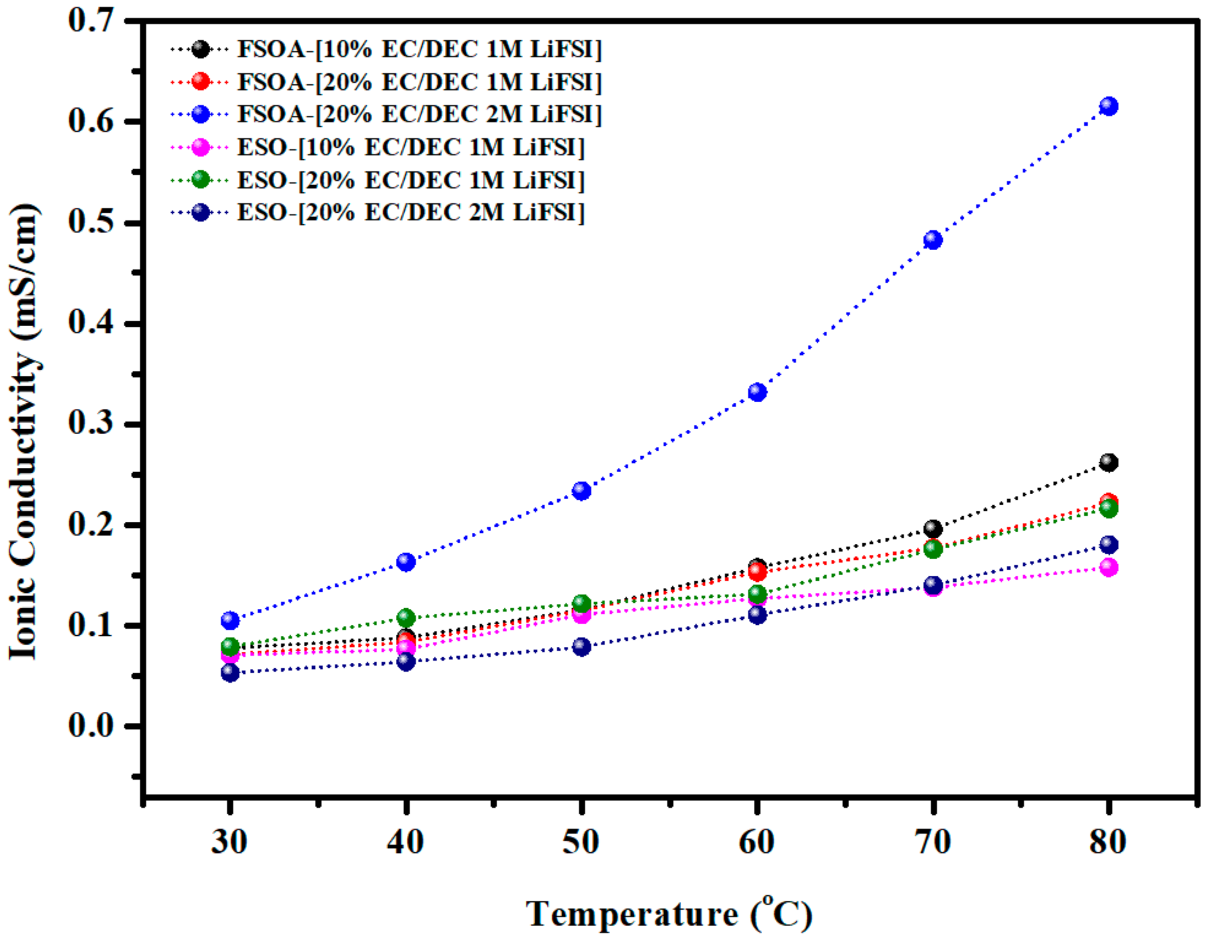

Figure S4. ionic conductivity *vs*. temperature plots of varied concentrated LiFSI and EC/DEC in FSOA (5:5 wt ratio) and ESO electrolytes.

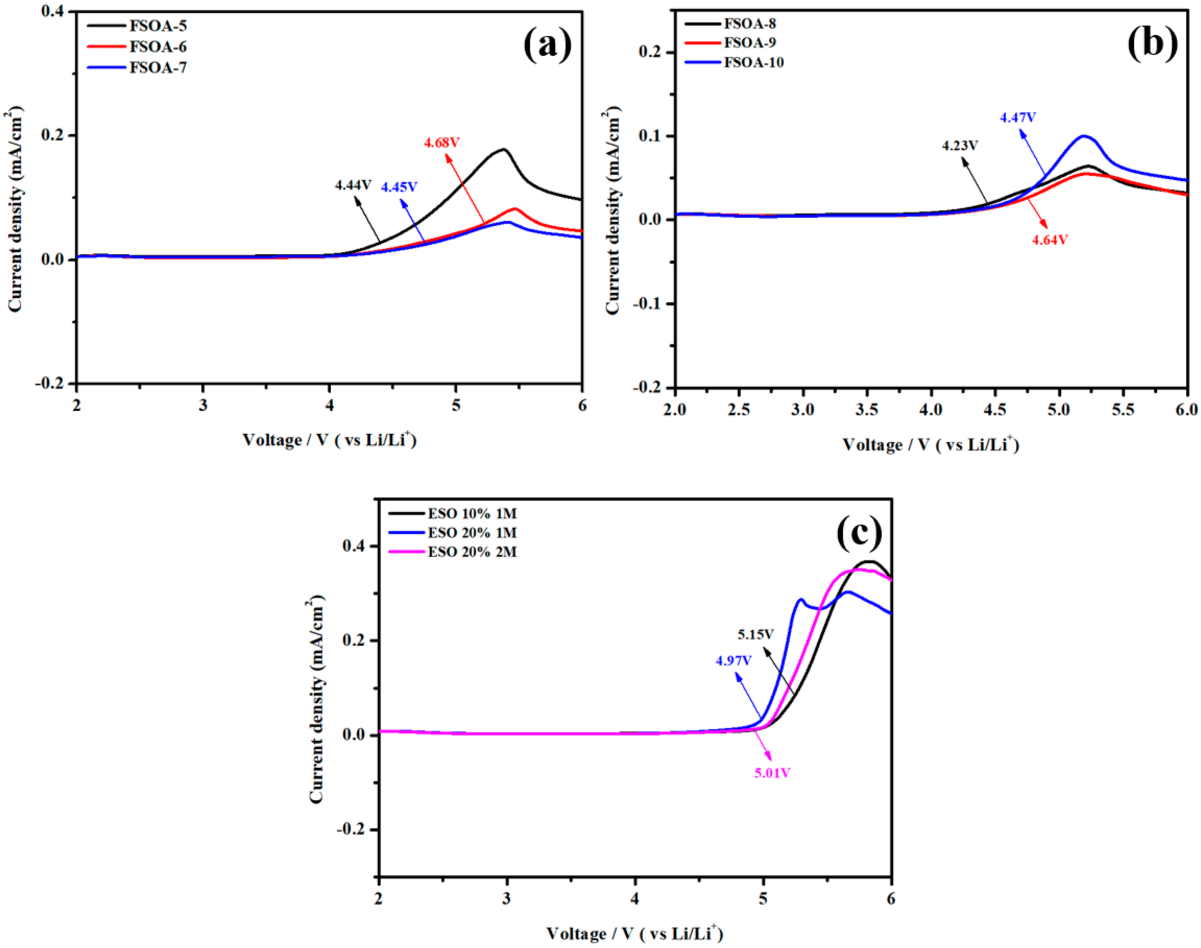

Figure S5. Linear sweep voltammetry (LSV) curves of (a) FSOA-5, FSOA-6, and FSOA-7, (b) FSOA-8, FSOA-9, and FSOA-10, and (c) ESO-based electrolytes with different salt concentrations, measured at room temperature with SS/electrolyte/Li cells.

Table S2. Lithium-ion transference numbers (t^{+}_{Li}) of FSOA- and ESO-based polymer electrolytes

Electrolyte name	I_0	I_s	R_0	R_s	ΔV	t_{Li}^{+}
FSOA-1	0.000013238	0.000007746	52.05	61.30	0.01 V (10 mV)	0.57
FSOA-2	0.000036741	0.00002366	40.98	48.46		0.62
FSOA-3	0.000015223	0.000009673	51.21	43.49		0.61
FSOA-5	0.0000291	0.000015925	90.05	86.37		0.47
FSOA-6	0.000036864	0.000021151	71.34	86.60		0.52
FSOA-7	0.000015507	0.000008984	84.86	84.90		0.54
ESO 10% 1M	0.000014683	0.000006292	61.63	73.82		0.41
ESO 20% 1M	0.000005943	0.000002358	78.49	79.55		0.39
ESO 20% 2M	0.000010253	0.000004669	52.40	49.43		0.44

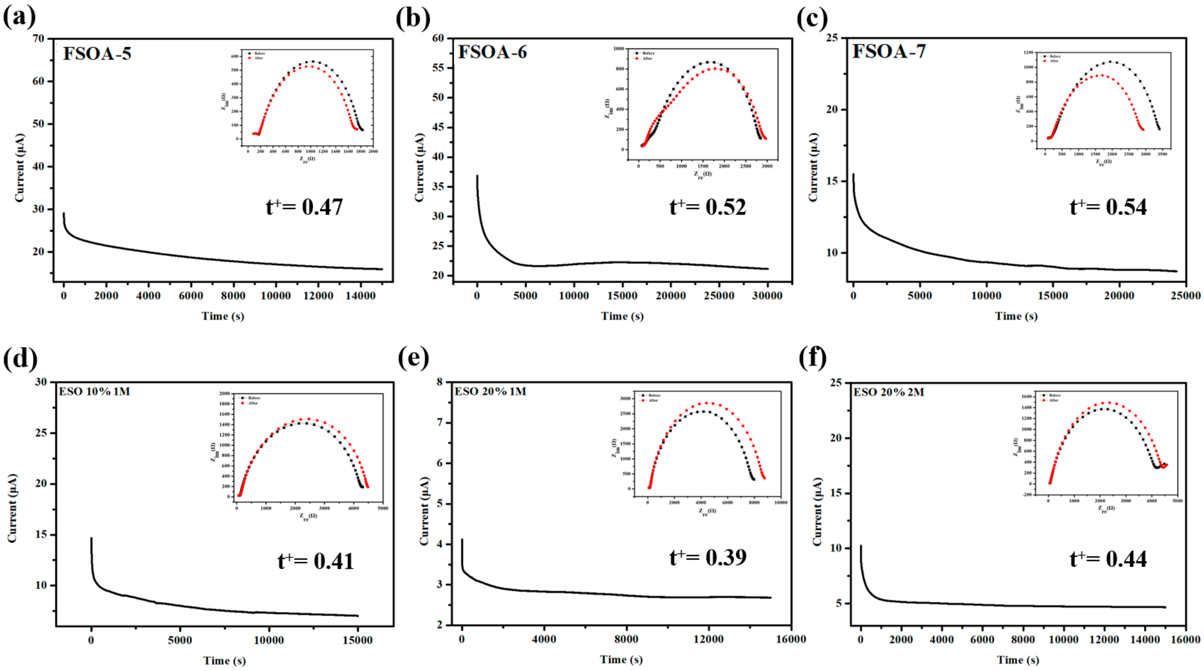

Figure S6. Chronoamperometry (CA) and impedance spectra before/after polarization plot of FSOAs and ESO-based electrolytes with different salt concentrations
